# A Geomedical Survey: Is There an Association Between Climatic Conditions and *Leishmania* Species Distribution in Iran During the Years 1999–2021?

**DOI:** 10.1007/s11686-024-00811-4

**Published:** 2024-02-28

**Authors:** Zahra Navi, Abdolreza Salahi-Moghaddam, Majid Habibi-Nokhandan, Mehdi Mohebali, Homa Hajjaran, Màrius V. Fuentes

**Affiliations:** 1 Department of Medical Parasitology and Mycology, School of Public Health, Tehran University of Medical Sciences, Poor Sina Avenue Qods ST, Keshavarz Blvd, 1417613151 Tehran, Iran; 2 Eco-Parasitologist, Aban Monife Institute of Public Health Research, Tehran, Iran; 3 Climatological Research Institute, Mashhad, Iran; 4 Parasites and Health Research Group, Departament de Farmàcia iI Tecnologia Farmacèutica i Parasitologia, Facultat de Farmàcia, Universitat de València, Av. Vicent Andrés Estellés s/n, Burjassot, 46100 València, Spain

**Keywords:** *Leishmania*, Climatic data, Epidemiology, GIS, Predicting map, Iran

## Abstract

**Purpose:**

Iran is among the high-risk leishmaniasis regions in the world. WHO recommends the use of GIS as an ideal tool for healthcare authorities to predict the evolution of a disease, delimit the risk of outbreaks and identify critical areas. The aim of this research is to find the association between the main species of *Leishmania* (*L. major*, *L. tropica*, *L. infantum*) dispersion and climatic variables in Iran.

**Methods:**

All molecular-based reports of leishmaniasis from Iran between 1999 and 2021 were gathered from reliable medical sources. Meteorological data (air and soil temperatures, annual rainfall and humidity) of the country along the study period were obtained from the Iranian Climatological Research Centre. The data concerning species distribution and climatic conditions during this period were moved to a base-map through raster layers using ArcGIS 10.4.1 software. The relationship between parasitological and climatic models was examined using ANOVA.

**Results:**

High risk area maps, based on the cut-off thresholds, were generated for *Leishmania major, L. tropica* and *L. infantum.* According to the molecular-based reports, the *L. major* distribution was significantly related to all climatic variables, while *L. tropica* was merely related to rainfall and humidity, and the *L. infantum* distribution was significantly associated with rainfall, soil and air temperatures.

**Conclusion:**

The association between climatic conditions and *Leishmania* species distribution in Iran has been confirmed. Consequently, both, the relationship between climatic conditions and the geographical distribution of *Leishmania* species, and the use of GIS to better understand the spatial epidemiology of leishmaniasis, have been reaffirmed.

**Supplementary Information:**

The online version contains supplementary material available at 10.1007/s11686-024-00811-4.

## Introduction

Leishmaniasis, a vector-borne parasitic disease, presents clinical manifestations which range from self-healing primary skin infections, in the case of cutaneous leishmaniasis, to incurable Kala-Azar, in the case of visceral leishmaniasis [1]. *Leishmania* parasites are mainly transmitted through zoonotic or anthroponotic routes, i.e. by bites of phlebotomine sandfly species [2]. Difficulties in leishmaniasis elimination and its control are rooted in poor vector (sandfly) management, the fact that there is no vaccine available, and the lack of new effective treatments [3].

The wide diversity of *Leishmania* species in 98 countries around the world has been documented. The scientific literature shows that about 350 million people worldwide are at risk of leishmaniasis, and an annual incidence of 700,000 to 1 million has been reported [4–7]. Although the actual number of human cases of cutaneous and visceral leishmaniasis is unknown, both have been expanding during the last decades [8–10]. Leishmaniasis shows a rising prevalence with a high incidence, especially in Mediterranean countries and across Europe [11]. As an example, the population of Barcelona (Spain) experienced a growing trend of leishmaniasis from 1996 to 2019, which was statistically significant between 2016 and 2019 [12]. Some researchers suggest that this obvious increase of vector borne diseases, such as leishmaniasis, might be due to climate change [13, 14]. Human health is affected by various environmental factors, especially in places of residence, so that it should be considered that most health issues have a spatial dimension [15]. In addition, some research has shown the influence of climate change on vector populations and, consequently, on the incidence of vector borne diseases, such as malaria, chikungunya, dengue, yellow fever, and zika [16, 17].

Geographic Information Systems (GIS) consist of hardware, software, geographic data, human resources, as well as other layers that display the results in the form of maps which can be analyzed for ecological and epidemiological aims [18]. Preparing a map of the occurrence of the disease at a particular point or region based on GIS, does not only protect communities of risk factors, but also influences the strategy of healthcare management [19]. Delimiting the risk of outbreaks and identifying critical areas by epidemiologists can open up new lines for healthcare authorities to create plans to prevent the spread of a disease [20]. WHO has recommended the use of GIS as an ideal tool to predict the future evolution of a disease in various areas and to analyse the relation between geographic health problems in communities and their natural environment [19].

The aims of this research are the following:

- to study the climatic conditions in the areas that are the centres of visceral and cutaneous leishmaniasis in Iran during the years 1999-2021;
- to identify climatic factors that increase the risk of an area turning into a hotspot region of cutaneous and visceral leishmaniasis; and to establish the association between the main species of *Leishmania* (*L. major*, *L. tropica*, *L. infantum*) dispersion and climatic variables.

## Materials and Methods

### Study Site

This study was conducted at a national scale in Iran, located in west Asia, covering a land mass of 1,648,195 km^2^, bordering the Caspian Sea in the north as well as the Persian Gulf and the Oman Sea in the south, and having borders with Afghanistan, Armenia, Azerbaijan, Iraq, Pakistan, Turkey and Turkmenistan [21].

### Data Collection

Parasitological data consisted of all molecular-based reports of leishmaniasis in Iran from 1999 to 2021, which were gathered from reliable medical sources. For the purpose of the current study, articles that report the number, type and geographical distribution of human leishmaniasis cases in Iran through molecular tests from 1999 to 2019 were collected based on a literature review previously published by our colleagues in 2021 [22]. We followed their method and added 11 new articles which were published from 2020 to 2021 to the database. To separate the reported data based on the cities located in each province, material and methods and results of all 168 articles were reviewed, and the results of 106 cities were recorded.

### Meteorological Data

The data of 382 meteorological stations around the whole country from 1999 to July 2021, including air temperature, soil temperature, annual rainfall and humidity, were obtained from the Iranian Climatological Research Centre.

All the above-mentioned data were arranged in a geodatabase for further application in ArcGIS.

### Mapping and Statistical Analysis

All the information available concerning the species distribution in Iran and climatic conditions during these 22 years was transported to maps through raster layers using ArcGIS 10.4.1. The maps generated were used for the proposed hypotheses concerning *Leishmania* spp. spatial epidemiology.

As the data were reported for certain locations only, the Inverse Distance Weighting (IDW) interpolation method was used to generate raster maps to estimate cutoff points for optimum climatic parameters, with the aim to predict the transmission risk in the regions of the country for which parasitological and climatic data were not available.

The relationship between *Leishmania* spp. foci and climatic models was statistically examined to determine the normal or abnormal distribution of data and quantitative data, which were then ranked to be selected for appropriate statistical analysis.

The relationship between parasitological data (dependent variable) and climatic models was examined with SPSS software version 20 using the ANOVA statistical test. A *P* value < 0.05 was considered statistically significant. Climatic conditions were grouped if necessary. Groups of ecological parameters in and out of the endemic area were estimated and the limits of statistical relationship with species distribution were investigated and recorded. The cut-off grouped boundaries were entered in the GIS project and the maps of high-risk areas were generated. For this purpose, changes were made in the grouping or some low-impact variables were basically omitted to obtain the optimal conditions that had the most geographical similarity with the reported conditions.

## Results

New molecular-based reports of the three main human *Leishmania* species in Iran from 2020 to 2021 are shown in Table 1, which were used, together with the previous reports from 1999 to 2019 [22], as part of the geodatabase in the GIS.

**Table 1:** Molecular-based reports of the three main human *Leishmania* species in Iran from 2020 to 2021

Province	City / Area	*L. tropica*	*L. major*	*L. infantum*	References
Yazd	Yazd	72	87	0	[23]
Fars	Shiraz	26	168	14	[24, 25]
Fars	Kharameh	11	70	0	[24]
Kerman	Kerman	50	0	0	[26]
Kerman	Bam	278	0	0	[27]
Sistan Balushestan	Chah bahar	54	6	0	[28]
Sistan Balushestan	Konarak	17	3	0	[28]
Khuzestan	Ahvaz	0	100	0	[29]
Sistan Balushestan	Zahedan	68	51	0	[30]
Tehran	Varamin	0	18	0	[31]
Khorasan North	Jajarm	0	629	0	[32]
Ghazvin	Qazvin	0	16	0	[33]

**Fig. 1:**
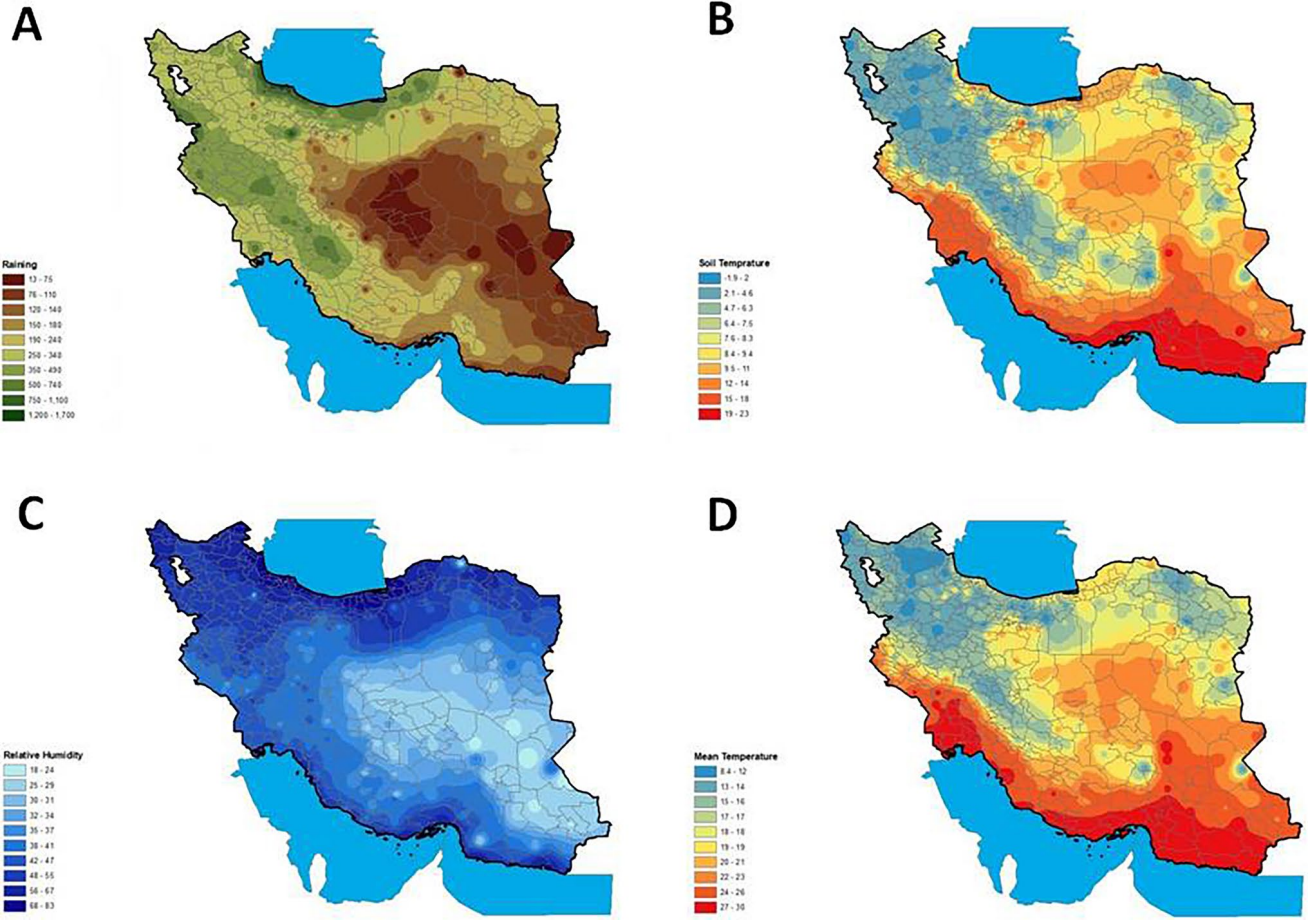
Interpolation at district resolution in Iran during the 1999–2021 period of: **A** annual rainfall (mm/year) (areas with brown colour and green colour have the lowest and highest annual rainfall, respectively); **B** soil temperature (℃) (red areas have the warmest and blue areas the coldest mean soil temperature); **C** relative humidity (%); **D** mean air temperature (℃) (red areas have the warmest and blue areas the coldest mean air temperature) (colour figure online)

For a better definition of the climatic conditions of the different parts of Iran at district resolution, meteorological data collected by synoptic stations were processed using the IDW prediction model in ArcGIS 10.4.1 software. Figure 1 shows the climatic conditions of the districts in Iran.

**Fig. 2:**
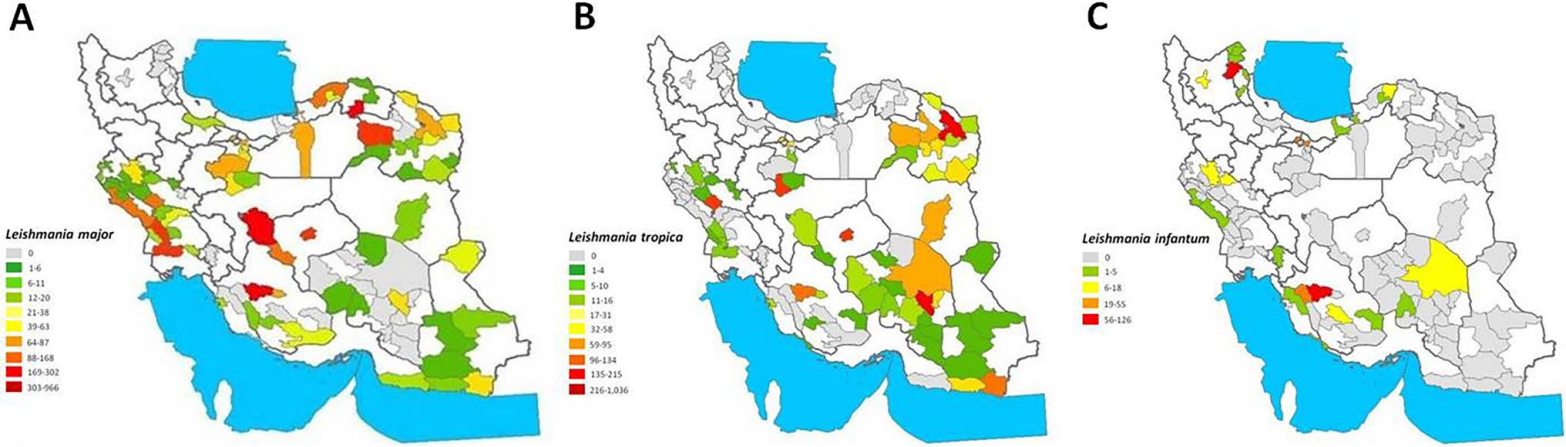
Geographical distribution recorded between 1999 and 2021 in Iranian cities of: **A** *Leishmania major* in 71 cities; **B** *Leishmania tropica* in 57 cities; **C** *Leishmania infantum* in 30 cities

**Fig. 3:**
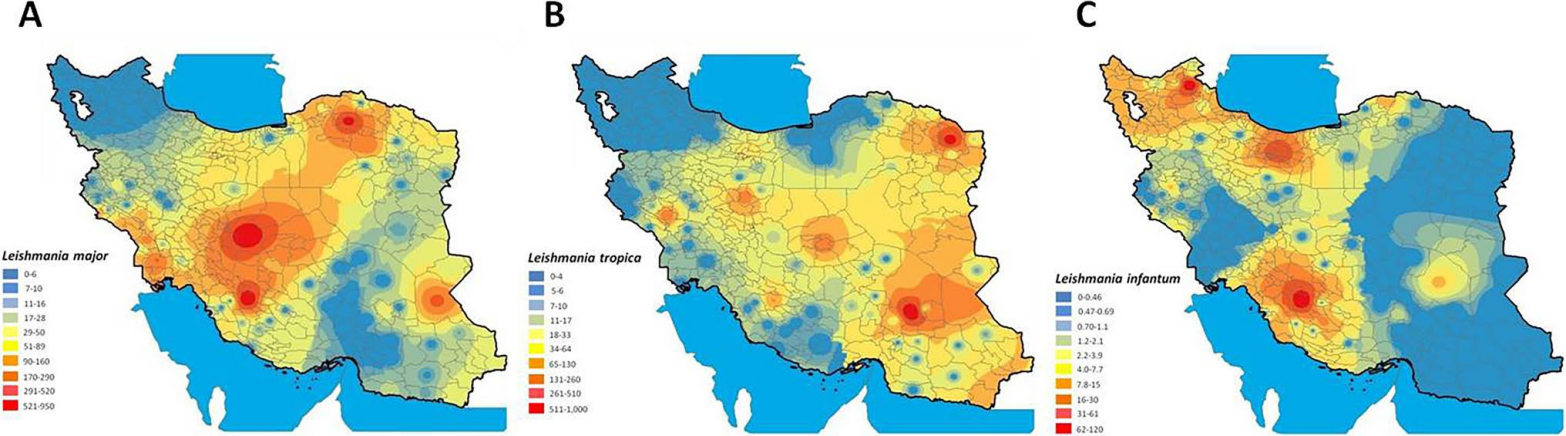
Distribution, using the IDW prediction model, in Iranian cities of: **A** *Leishmania major*; **B** *Leishmania tropica*; (C) *Leishmania infantum*

The data of the three *Leishmania* species reported at national scale from several locations were gathered and located on the maps (Fig. 2). The interpolation of these data was done by IDW showing the pattern of *L. major*, *L. tropica* and *L. infantum* (Fig. 3).

The climatic conditions of the high prevalence areas of the three *Leishmania* species were statistically analysed (Supplementary Table 1, 2 and 3; Supplementary Figs. 1, 2 and 3). The results show that some climatic parameters do not have an important impact on the parasite distribution and therefore do not contribute to the epidemiology of leishmaniasis; consequently, they were not considered in the prediction models.

In the next step, suitable climatic conditions were arranged in lower and higher groups; these cutoff conditions were tested using ANOVA in SPSS software. A correlation with a *P* value < 0.05 was assumed as statistical difference to omit some climatic parameters.

The distribution of *L. major* in Iran is significantly related to: the annual rainfall (*P* value = 0.004), with the optimum for its distribution being about 175 to 225 mm per year; the soil temperature (*P* value < 0.0001), with the optimum being about 8 to 11 ℃; the relative humidity (*P* value < 0.0001), with the optimum being about 37 to 40%; and the mean-minimum and maximum air temperature (*P* value < 0.05), with the mean optimum being about 15 to 18 ℃.

The distribution of *L. tropica* significantly related to: the annual rainfall (*P* value = 0.003), with the optimum for its distribution being about 150 to 200 mm per year; and the relative humidity (*P* value = 0.008), with the optimum being about 37 to 40%.

The distribution of *L. infantum* in Iran is significantly related to: the annual rainfall (P value < 0.0001), with the optimum for its distribution being about 250 to 350 mm per year; the soil temperature (*P* value < 0.0001), with the optimum being about 7 to 8 ℃; and the mean-minimum and maximum air temperature (*P* value < 0.05), with the optimum being about 13 to 15 ℃.

Suitable climatic conditions of ecological niches of the three main *Leishmania* species in Iran are:

- 95-325 mm of annual rainfall, 5–16 ℃ of soil temperature, 31–50% of relative humidity, 15–27 ℃ of mean temperature, 21–34 ℃ of maximum temperature, and 8–19 ℃ of minimum temperature, in the case of *L. major*;
- 95–210 mm of annual rainfall, and 25–47% or relative humidity, in the case of *L. tropica*;
- 250–350 mm of annual rainfall, 4–8 ℃ of soil temperature, and 520 of mean temperature, in the case of *L. infantum*.

**Fig. 4:**
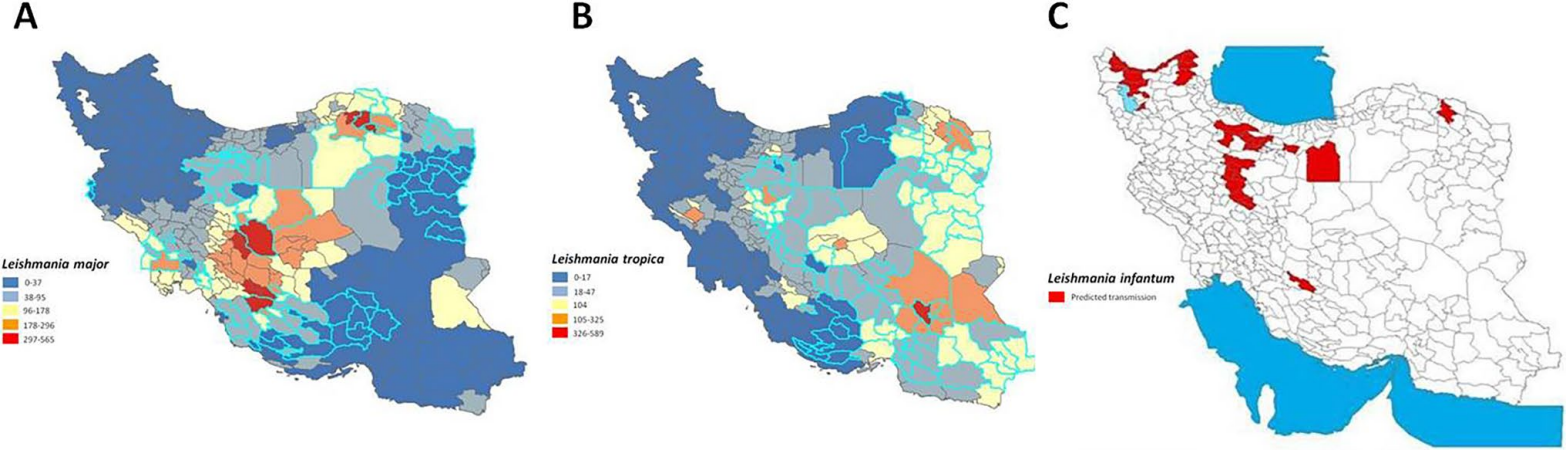
Transmission predicted by the climatic model of: **A** *Leishmania major*; **B** *Leishmania tropica*; **C** *Leishmania infantum*

These critical conditions were assumed, and predictive maps were generated for each species. The maps show the transmission of leishmaniasis predicted by the climatic models for *L. major*, *L. tropica* and *L. infantum* (Fig. 4). Unfortunately, the *L. infantum* prediction map could not be developed in more detail as for the other two species due to its more reduced area of distribution and data were only recorded in 30 cities of Iran.

## Discussion

Leishmaniasis is well established in Iran and almost all reports to health centres reflect the local disease scenario, with the exception of the capital city, Tehran. Cases diagnosed and reported in Tehran are not of local transmission and in prediction of the ecological niche of parasites this epidemiological item was considered a correction.

It is notable that other epidemiological parameters are stand-alone factors in the distribution of leishmaniasis, for instance, an urban area for *L. tropica* or the presence of dogs for the distribution of *L. infantum*.

*Leishmania major* is the species whose distribution has been most closely related to climatic factors. All ecological variables examined in the study were significantly associated with *L. major* distribution in Iran. Surprisingly, mapping of *L. major* cases according to molecular reports during 1999–2021 shows that their geographic distribution is limited, stretching from the northeast of Iran to the centre and to the southwest of the country. The main reservoir of *L. major* in the northeast and centre of Iran is the rhombic rat, *Rhombus opimus*, and the secondary reservoir in these areas is the Libyan jird, *Meriones libycus*, while the main reservoir in the southwest of Iran is the Indian gerbil, *Tatera indica* [34, 35]. As the rodent reservoir and the parasite distributions are largely coincident, it can be concluded that *L. major* is indirectly affected by climate factors. This conclusion is highly in agreement with its life cycle and its transmission route which is mainly dependent on the rodent population [36]. In contrast, *L. tropica* prevalence is only related to humidity and annual rainfall. This lack of dependence on air and soil temperatures might be an important reason for its geographical distribution, which has spread throughout the country except for a small area in the northwest. It is obvious that the complete independence of *L. tropica* from air and soil temperatures may lead to an easy outbreak, and therefore, the best way for its control and management seems to be the early diagnosis and treatment of patients. In the case of *L. infantum*, its prevalence is related to all climatic variables considered with the exception of humidity. In comparison to *L. major*, *L. infantum* prevalence tends to be more closely linked to colder and rainier geographical regions, i.e. to cold and rainy locations.

Periodical examination of dogs for visceral leishmaniasis seems to be unavoidable. Obviously, given the fatal complications of visceral leishmaniasis, if sufficient attention is not paid to the early diagnosis and treatment of dogs, the main reservoir of this zoonotic disease, in areas with a suitable climate, the consequences will be costly to both animal and human health.

## Conclusions

The current results of the 1999–2021 study period confirm the association between climatic conditions and *Leishmania* species distribution in Iran, especially in the case of *L. major* and *L. infantum*, whilst being less significant in the case of *L. tropica*. Consequently, the relationship between climatic conditions and the geographical distribution of *Leishmania* species as well as the use of GIS as an essential tool to better understand the spatial epidemiology of leishmaniasis have been reaffirmed.

### Supplementary Information

Below is the link to the electronic supplementary material.

Supplementary file1 Fig. 1 (A) Annual rainfall (mm); (B) Soil temperature (℃); (C) Relative humidity (%); (D) Mean temperature (℃); in the *Leishmania major* high prevalence areas (JPG 56 KB)

Supplementary file2 Fig. 2 (A) Annual rainfall (mm); (B) Relative humidity (%); in the *Leishmania tropica* high prevalence areas (JPG 27 KB)

Supplementary file3 Fig. 3 (A) Annual rainfall (mm); (B) Soil temperature (℃); (C) Mean temperature (℃); in the *Leishmania infantum* high prevalence areas (JPG 37 KB)

Supplementary file4 (DOCX 24 KB)

## Data Availability

The database used to carry out the present study is not publicly available due to our departments’ internal policy. However, the database could be made available, after a justification of its use, upon request from the corresponding author.
