# Supplementary figures and images for: A Geomedical Survey: Is There an Association Between Climatic Conditions and Leishmania Species Distribution in Iran During the Years 1999–2021?
Source: Acta Parasitol. 2024 Feb 28;69(1):769–75. doi: 10.1007/s11686-024-00811-4 (PMC11001653; doi:10.1007/s11686-024-00811-4)

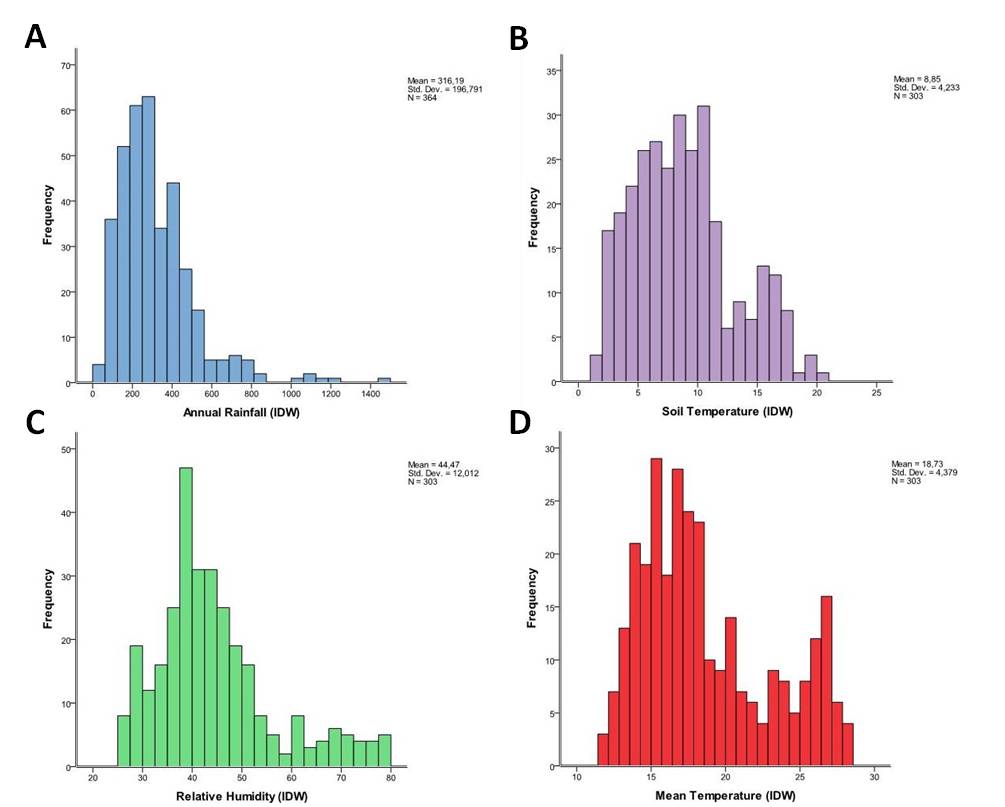

Supplement: Supplementary file 1 — Supplementary file1 Fig. 1 (A) Annual rainfall (mm); (B) Soil temperature (℃); (C) Relative humidity (%); (D) Mean temperature (℃); in the Leishmania major high prevalence areas (JPG 56 KB) [file 11686_2024_811_MOESM1_ESM.jpg]

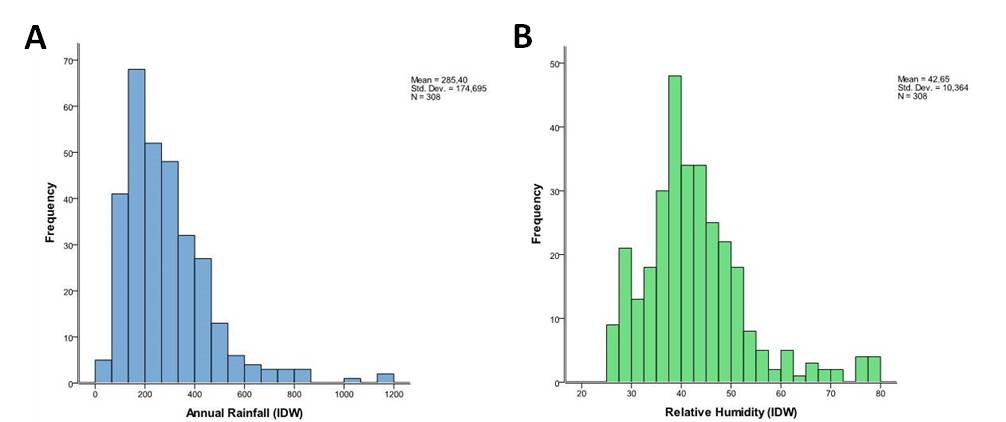

Supplement: Supplementary file 2 — Supplementary file2 Fig. 2 (A) Annual rainfall (mm); (B) Relative humidity (%); in the Leishmania tropica high prevalence areas (JPG 27 KB) [file 11686_2024_811_MOESM2_ESM.jpg]

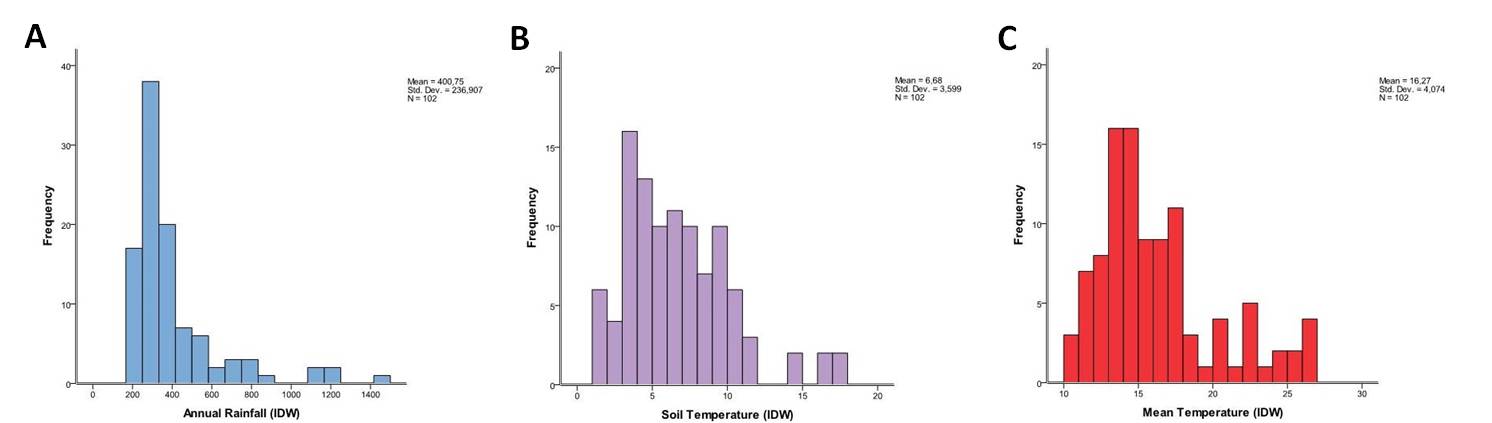

Supplement: Supplementary file 3 — Supplementary file3 Fig. 3 (A) Annual rainfall (mm); (B) Soil temperature (℃); (C) Mean temperature (℃); in the Leishmania infantum high prevalence areas (JPG 37 KB) [file 11686_2024_811_MOESM3_ESM.jpg]
