# Supplementary material for: A Geomedical Survey: Is There an Association Between Climatic Conditions and Leishmania Species Distribution in Iran During the Years 1999–2021?
Source: Acta Parasitol. 2024 Feb 28;69(1):769–75. doi: 10.1007/s11686-024-00811-4 (PMC11001653; doi:10.1007/s11686-024-00811-4)
Supplement: Supplementary file 4 — Supplementary file4 (DOCX 24 KB) [file 11686_2024_811_MOESM4_ESM.docx]

**Supplementary Table 1** Climatic conditions in the geographical areas where *Leishmania major* is highly prevalent (prevalence higher than the average overall prevalence in the country) (n = 364)

|  | **Annual Rainfall (mm)** | **Soil Temperature (ºC)** | **Relative Humidity (%)** | **Mean Temperature (ºC)** | **Minimum Temperature (ºC)** | **Maximum Temperature (ºC)** |
| --- | --- | --- | --- | --- | --- | --- |
| Mean | 316.19 | 8.78 | 44.98 | 18.67 | 11.52 | 24.65 |
| Median | 274.24 | 8.43 | 42.688 | 17.52 | 10.79 | 23.39 |
| Std. Deviation | 169.79 | 4.531 | 11.99 | 4.574 | 4.46 | 4.650 |
| Minimum | 47.70 | 0.70 | 26.22 | 11.11 | 3.80 | 17.06 |
| Maximum | 1478.63 | 20.97 | 80.32 | 28.42 | 23.01 | 33.98 |

**Supplementary Table 2** Climatic conditions in the geographical areas where *Leishmania tropica* is highly prevalent (prevalence higher than the average overall prevalence in the country) (n = 308)

|  | **Annual Rainfall (mm)** | **Soil Temperature (ºC)** | **Relative Humidity (%)** | **Mean Temperature (ºC)** | **Minimum Temperature (ºC)** | **Maximum Temperature (ºC)** |
| --- | --- | --- | --- | --- | --- | --- |
| Mean | 285.40 | 9.04 | 42.65 | 19.09 | 11.74 | 25.17 |
| Median | 247.28 | 8.45 | 41.13 | 17.59 | 10.79 | 23.66 |
| Std. Deviation | 174.70 | 4.632 | 10.36 | 4.675 | 4.60 | 4.69 |
| Minimum | 47.70 | 1.48 | 26.22 | 11.57 | 4.40 | 17.06 |
| Maximum | 1195.70 | 20.97 | 78.24 | 28.42 | 23.01 | 33.98 |

**Supplementary Table 3** Climatic conditions in the geographical areas where *Leishmania infantum* is highly prevalent (prevalence higher than the average overall prevalence in the country) (n = 102)

|  | **Annual Rainfall (mm)** | **Soil Temperature (ºC)** | **Relative Humidity (%)** | **Mean Temperature (ºC)** | **Minimum Temperature (ºC)** | **Maximum Temperature (ºC)** |
| --- | --- | --- | --- | --- | --- | --- |
| Mean | 400.75 | 6.68 | 51.12 | 16.27 | 9.72 | 21.83 |
| Median | 314.71 | 6.08 | 50.40 | 15.10 | 9.04 | 20.41 |
| Std. Deviation | 236.90 | 3.599 | 12.04 | 4.074 | 3.60 | 4.30 |
| Minimum | 191.55 | 1.17 | 30.05 | 10.43 | 4.16 | 15.81 |
| Maximum | 1478.63 | 17.92 | 80.32 | 26.93 | 20.78 | 33.03 |
